# Selective Functionalisation of 5‐Methylcytosine by Organic Photoredox Catalysis

**DOI:** 10.1002/anie.202304756

**Published:** 2023-05-15

**Authors:** Mathew M. Simpson, Ching Ching Lam, Jonathan M. Goodman, Shankar Balasubramanian

**Affiliations:** ^1^ Yusuf Hamied Department of Chemistry University of Cambridge Lensfield Road CB2 1EW Cambridge UK; ^2^ Cancer Research UK Cambridge Institute Li Ka Shing Centre University of Cambridge Robinson Way CB2 0RE Cambridge UK; ^3^ School of Clinical Medicine University of Cambridge CB2 0SP Cambridge UK

**Keywords:** C−H Functionalization, Nucleic Acids, Photocatalysis, Radicals, Reaction Mechanisms

## Abstract

The epigenetic modification 5‐methylcytosine plays a vital role in development, cell specific gene expression and disease states. The selective chemical modification of the 5‐methylcytosine methyl group is challenging. Currently, no such chemistry exists. Direct functionalisation of 5‐methylcytosine would improve the detection and study of this epigenetic feature. We report a xanthone‐photosensitised process that introduces a 4‐pyridine modification at a C(sp^3^)−H bond in the methyl group of 5‐methylcytosine. We propose a reaction mechanism for this type of reaction based on density functional calculations and apply transition state analysis to rationalise differences in observed reaction efficiencies between cyanopyridine derivatives. The reaction is initiated by single electron oxidation of 5‐methylcytosine followed by deprotonation to generate the methyl group radical. Cross coupling of the methyl radical with 4‐cyanopyridine installs a 4‐pyridine label at 5‐methylcytosine. We demonstrate use of the pyridination reaction to enrich 5‐methylcytosine‐containing ribonucleic acid.

## Graphical abstract

Selective functionalisation of the C(sp^3^)−H bond in the methyl group of 5‐methylcytosine is achieved by xanthone‐catalysed photoredox catalysis. The specific nature of the functionalisation of 5‐methylcytosine on ribonucleic acid (RNA) was used to install a Halo‐Tag reactive handle which enabled the chemical enrichment of 5‐methylcytosine containing RNA.

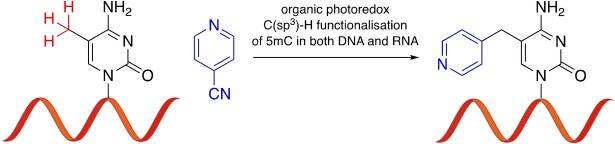

## Introduction

Methylation at the C5 position of cytosine to produce 5‐methylcytosine is a prevalent chemical modification in both ribonucleic acid (RNA) and deoxyribonucleic acid (DNA).[^1^, ^2^] Bisulfite sequencing (BS‐seq) has been the benchmark method to map 5‐methylcytosine in DNA (5mC) and RNA (m5C).[^3^, ^4^] Bisulfite treatment deaminates cytosine (C) to uracil (U), whereas 5mC deaminates 100 times slower.^[5]^ BS‐seq is highly destructive, particularly towards RNA.^[6]^ Improved methods to detect cytosine methylation in RNA are needed to build better understanding about the function of this modification. 5mC in messenger RNA (mRNA) is enriched at 5′ and 3′ untranslated regions of mRNAs in a tissue‐specific manner, suggestive of a posttranscriptional regulatory role.^[7]^

**Figure 1:**
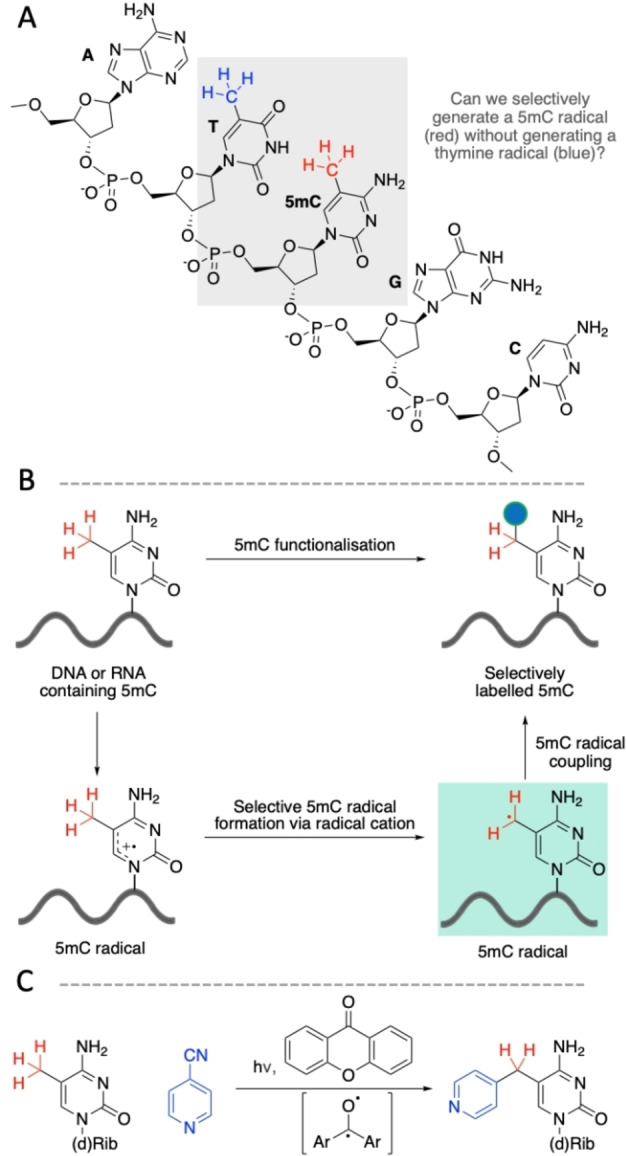
Strategy for selective modification of 5‐methylcytosine. (A) Canonical bases and 5mC. (B) Plan for covalent modification of 5mC via coupling the selectively generated 5mC radical with an appropriate partner. (C) Scheme of overall pyridination strategy.

The methyl group of 5mC is a distinct chemical moiety. The identification of chemical methods that selectively label the C5‐methyl group of 5mC would be valuable to help elucidate this vital epigenetic feature. This challenge demands selective targeting of the C(sp^3^)−H bond in the methyl group of 5mC over a plethora of other potential sites. We have explored whether selective 5mC‐methyl radical formation could enable a viable pathway to label 5mC. A key requirement for DNA is selectivity for the C5‐methyl group of 5mC over the C5‐methyl group of the canonical thymine (T) nucleobase (Figure 1A). The bond dissociation energies of the C−H bond in C5‐methyl groups of 5mC and T differ by less than 5 kJ mol^−1^ (387.3 kJ mol^−1^ and 383.1 kJ mol^−1^ respectively),^[8]^ therefore it would be challenging to achieve selectivity via 5mC‐methyl radical formation by direct hydrogen‐atom abstraction. 5mC‐methyl radical formation can also be achieved via deprotonation of the 5mC radical cation. The T nucleobase has a higher redox potential compared to 5mC by up to 0.53 V (Table S1, Supporting Information). We therefore reasoned that single electron transfer would be more favourable at the 5mC nucleobase, thereby providing a pathway to selectivity. We pursued the use of triplet photosensitisers capable of abstracting an electron from 5mC by single electron transfer.[^9^, ^10^] We explored a diverse range of sensitisers with a view to discovering the optimal sensitiser, based on its triplet excited state redox potential relative to the redox potential of 5mC.

We sought to trap the 5mC‐methyl radical by a compatible coupling partner (Figure 1B). The ideal coupling partner must be able to accept an electron from the photosensitiser radical anion following the single‐electron oxidation of 5mC to complete the catalytic cycle. The 4‐pyridination of simple benzylic radicals has been achieved using the triplet photosensitiser benzophenone and 4‐cyanopyridine (4‐CP).^[11]^ 4‐CP serves both as a benzylic radical coupling partner and regenerates the photosensitiser. We inferred that 4‐CP would be capable of coupling to a 5mC radical. We used density functional theory (DFT) and molecular mechanics (MM) calculations to propose a plausible mechanistic pathway.

**Figure 2:**
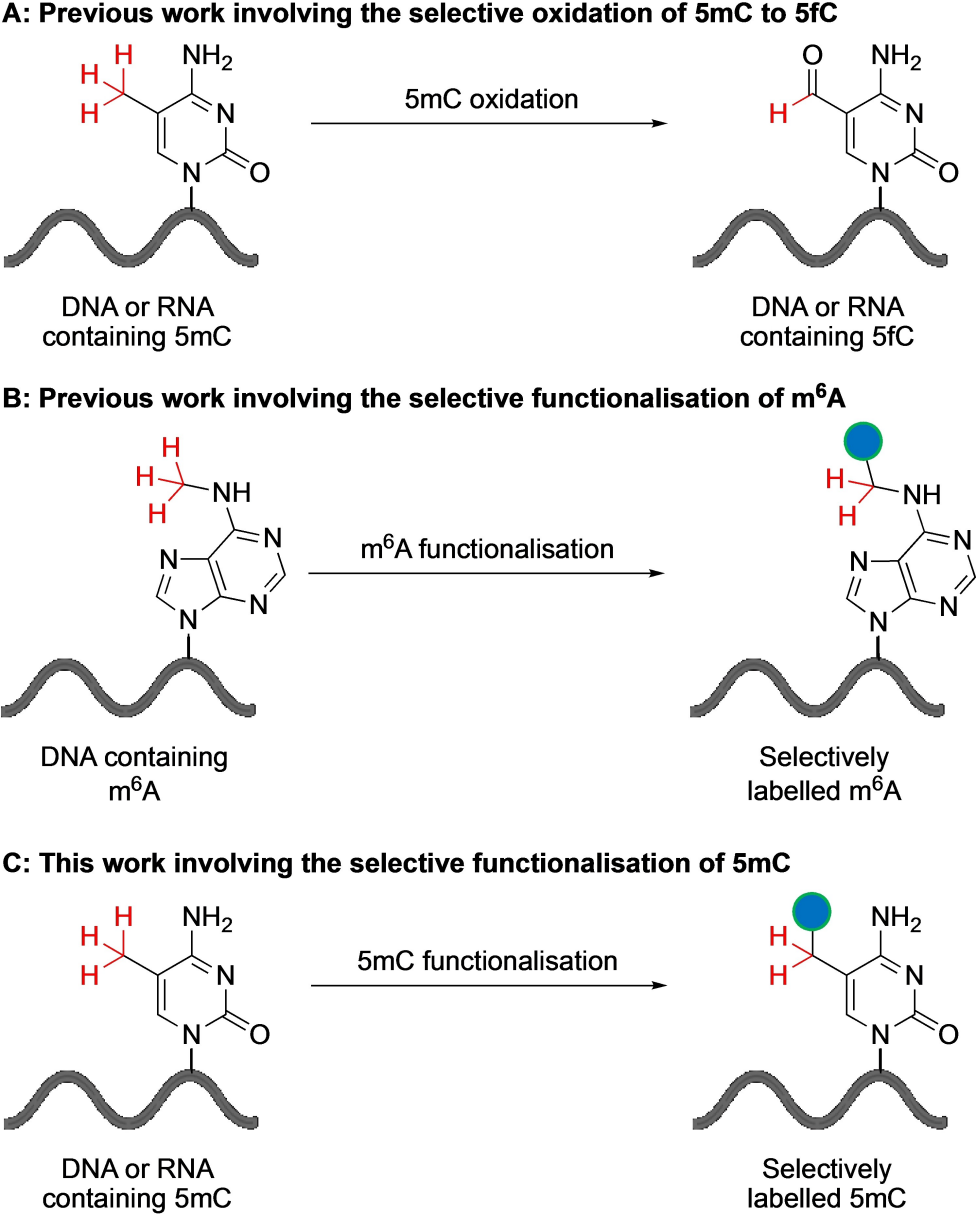
Studies involving the selective functionalisation of radicals in nucleic acids. A) Selective oxidation of 5mC to 5fC can be used to map 5mC using reaction that leads to the targeted degradation of 5fC or 5fC‐to‐T conversion. B) Selective functionalisation of m^6^A with a handle for chemical enrichment. C) This study involves the selective functionalisation of 5mC with a handle for chemical enrichment.

This work builds on the functional generation of radicals in nucleic acids reported by our group and others. For example, the one electron photooxidation of 5mC by 2‐methyl‐1,4‐naphthoquinone results in the formation of a 5mC‐methyl radical.^[9]^ In the presence of oxygen this leads to the oxidation of 5mC to 5‐formylcytosine (5fC) (Figure 2A). This has been proposed as a method to map 5mC in genomic DNA by targeted piperidine cleavage at the newly generated 5fC base.^[10]^ The biomimetic C−H oxidation of the 5mdC nucleoside was recently reported.^[12]^ Enzymatic oxidation of 5mC is preferred over chemical oxidation for 5mC detection as it is less destructive and more efficient.^[13]^ We have previously reported the selective functionalisation of N6‐methyladenosine (m^6^A) in DNA with an alkyne‐handle that enables chemical enrichment (Figure 2B).^[14]^ This involved the selective hydrogen‐atom abstraction at the N6‐methyl group catalysed by visible‐light‐mediated photoredox catalysis.

As our approach does not involve the overall oxidation of 5mC, we predict pyridination would be able to distinguish 5mC over its oxidised derivatives such as 5‐hydroxymethylcytosine (5hmC). Herein we present the chemical functionalisation of 5mC using 4‐CP and xanthone which selectively labels 5mC with a 4‐pyridine group at both the nucleoside and oligonucleotide level. We demonstrate how the specific nature of the pyridination reaction can be exploited to chemically enrich m5C‐containing RNA.

## Results and Discussion

We set out to selectively functionalize the C(sp^3^)−H of the 5mC methyl group by first identifying a photosensitiser capable of abstracting an electron from 5mC. We selected the photosensitiser xanthone over benzophenone for our study due to its larger triplet excited state reduction potential (*E* ^red^=+1.71 V vs Normal Hydrogen Electrode (NHE)) compared to benzophenone (*E* ^red^=+1.42 V vs NHE).^[15]^ The ground state redox potential of xanthone (*E* ^red^=−1.51 V vs NHE) is sufficiently negative to facilitate the single electron reduction of 4‐CP (*E* ^red^=−1.33 V vs NHE).^[16]^

While we were interested in both DNA and RNA, our initial studies focused on identifying conditions that resulted in the 4‐pyridination of the 5‐methyl‐2′‐deoxycytidine (5mdC) nucleoside. As 2′‐deoxythymidine (dT) also contains a methyl group we aimed to explore the potential for selective modification of 5mdC over dT. Irradiation was carried out at 365 nm as this wavelength is close to the absorbance maximum of xanthone, and is not damaging to DNA.^[17]^ Acetonitrile was selected as an organic cosolvent in order to solubilize the xanthone photocatalyst as well as 4‐CP. Oxygen was excluded from the reaction in order to minimize undesirable oxidation of 5mdC. Upon irradiation with near‐UV light (365 nm) under argon (Ar) at room temperature, 4‐pyridine‐functionalised 5mdC (4‐Pyr‐5mdC) was obtained in 42 % yield, as judged by tandem liquid chromatography‐mass spectrometry (LC–MS) analysis (Entry 1, Table 1). Detectable levels of 5‐hydroxymethyl‐2′‐deoxycytidine (5hmdC) and 5‐formyl‐2′‐deoxycytidine (5fdC) bi‐products were observed when the reaction was performed under atmospheric conditions while the yield of 4‐Pyr‐5mdC was similar (Entry 7 Table 1). We then investigated the selectivity of the pyridination reaction on other common DNA nucleosides. Only a 3 % yield of 4‐pyridination derivative was observed on the dT nucleoside (Entry 2, Table 1). There was no detectable reactivity of 2′‐deoxycytidine (dC), 2′‐deoxyguanosine (dG) and 2′‐deoxyadenosine (dA) towards pyridination (Entries 3–5, Table 1). Therefore, the pyridination reaction conditions using xanthone exhibits good selectivity for 5mdC. With a triplet excited state redox potential of +1.71 V vs NHE, xanthone can abstract an electron from all the canonical nucleosides. dG, dA, dC and dT have redox potentials of +1.29 V, +1.42 V, +1.60 V and +1.70 V respectively.^[18]^ The inertness of dC, dG and dA may be attributed to the fact that in the absence of oxygen the only available reaction pathway is back electron transfer from the xanthone radical anion to the nucleoside radical cation.^[10]^ To validate this hypothesis, we carried out the nucleoside pyridination reaction under atmospheric conditions. The presence of oxygen reduced the recovery of all four canonical nucleosides (Entries 8–11, Table 1). It is likely single electron transfer occurs on all the nucleosides, but only those with a benzylic‐like methyl group can react with 4‐CP.

**Table 1:** Pyridination of DNA Nucleosides.

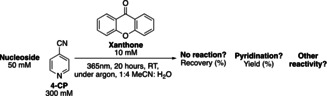			
Entry	Nucleoside	Unreacted Nucleoside [%]^[a]^	Yield [%]^[a]^
1(Ar)	5mdC	7	42
2(Ar)	dT	67	3
3(Ar)	dC	99	0
4(Ar)	dG	97	0
5(Ar)	dA	94	0
6(Ar)	5hmdC	34	3
7(air)	5mdC	19	43
8(air)	dT	29	3
9(air)	dC	88	0
10(air)	dG	57	0
11(air)	dA	51	0
12(air)	5hmdC	1	3
13(Ar,dark)	5mdC	100	0
1(Ar)	5mdC	7	42
2(Ar)	dT	67	3

[a] Percentage recovery and yields were calculated by integrating the UV absorbance of LCMS peaks relative to 2′‐deoxyinosine internal standard.

The presence of the methyl group in 5mdC increases the pyrimidine ring electron density, thus 5mdC is expected to have a lower oxidation potential relative to dC.^[19]^ The difference in redox potentials of 5mdC and dT may contribute to the selectivity for 5mdC over dT towards pyridination. In order to further support the observed selectivity for 5mdC over dT we performed DFT calculations at the B3LYP‐D3/6‐31G(d)//ωB97X‐D/6‐311++G(d,p) level of theory.[^20^, ^21^, ^22^, ^23^, ^24^] The calculations show that the Gibbs free energy change for single electron transfer to triplet excited xanthone is +10.0 kcal mol^−1^ greater for dT as compared to 5mdC. Therefore, single electron oxidation by xanthone is more favourable for 5mdC than for dT.

Only a 3 % yield of pyridination was observed when the pyridination reaction was performed on the 5hmdC nucleoside (Entry 6, Table 1). Significant oxidation of 5hmdC to 5fdC was observed. 34 % of 5hmdC was unreacted following the pyridination reaction, the remaining 63 % was oxidised to 5fdC (Figure S7, Supporting Information). The oxidation of 5hmdC to 5fdC under the pyridination conditions was an unexpected result in the absence of oxygen. The exact mechanism of 5hmdC oxidation is unknown, however it appears to outcompete the pyridination pathway. As the yield of pyridination of 5mdC is substantially higher than that of 5hmdC, xanthone‐photosensitised 4‐pyridination has the potential to discriminate 5mdC from 5hmdC in biochemical applications.

**Figure 3:**
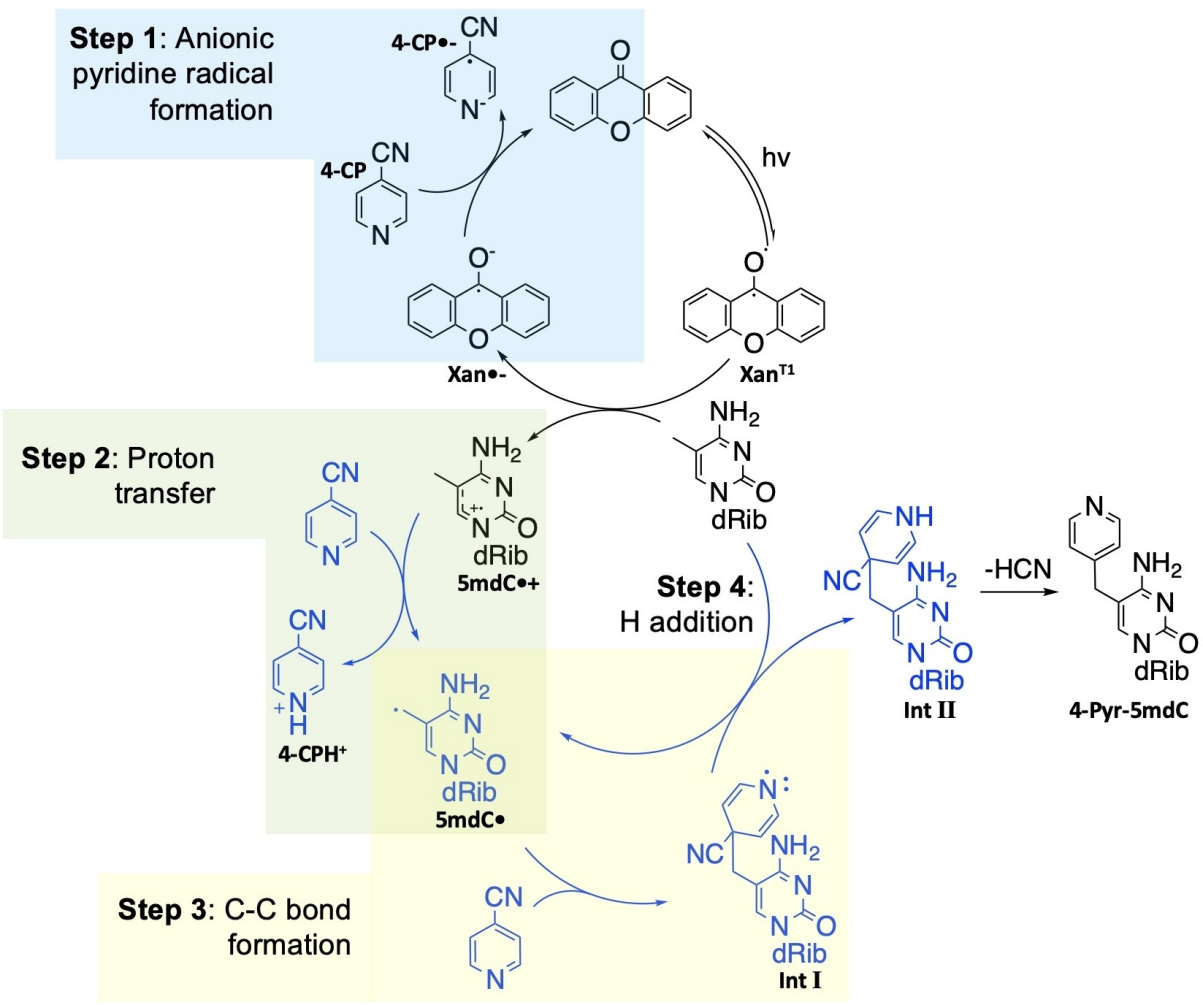
The proposed pyridination reaction mechanism. The steps in black have been proposed in previous studies for pyridination reactions on benzylic C(sp^3^)−H bonds by Inoue et al. Instead of this radical‐radical mechanism, we propose the pathway in grey for the proton transfer and C−C bond formation.

The proposed mechanism for the reaction (Figure 3) was tested with DFT calculations on possible competing pathways (Section 5.2–5.4, Supporting Information). This differs from previous mechanistic proposals for similar reactions, which suggest a radical‐radical coupling as the key carbon‐carbon bond forming step (Figure S36A, Supporting Information).[^9^, ^10^, ^21^, ^22^, ^23^, ^24^] Our kinetic studies suggest that Δ*G* ^≠^ for C−C bond formation by either radical‐radical coupling or by addition of 5mdC⋅ to 4‐CP are comparable (Section 5.3.2, Supporting Information). The concentration of radicals in the reaction mixture should be low due to the catalytic amount of xanthone used and so the chance of a collision between two radicals is also low. Xanthone in a triplet excited state Xan^T1^ abstracts an electron from 5mdC by single‐electron transfer, generating the 5mdC radical cation 5mdC⋅^+^ and the xanthone radical anion Xan⋅^−^. The xanthone radical anion subsequently reduces 4‐CP to the radical anion 4‐CP⋅^−^ and regenerates xanthone in the ground state (Step 1, Figure 3). The neutral 5mdC radical 5mdC⋅ is most likely generated via proton transfer from 5mdC⋅^+^ to 4‐CP (Step 2, Figure 3). C−C bond is then formed between a neutral pyridine and 5mdC⋅ (Step 3, Figure 3). The preferred pathway for arriving at IntII is via hydrogen‐atom abstraction from a neutral 5mdC molecule to the adduct IntI (Step 4, Figure 3 & Section 5.3.3, Supporting Information). Finally, the elimination of HCN completes the formation of the pyridination product 4‐Pyr‐5mdC.

As a step towards harnessing this 5mC‐specific pyridination chemistry for applications, we explored whether derivatives of 4‐CP could function as suitable substrates in the pyridination reaction. With a pyridination yield of 58 %, 3‐methyl‐4‐cyanopyridine (3‐Me‐4‐CP) was a better substrate than 4‐CP (Entry 2, Table 2). 3‐fluoro, 3‐chloro, 3‐cyano, and 3‐hydroxy substitutions were all acceptable substrates for pyridination (Entries 3–7, Table 2). No pyridination product was observed for 3‐amino‐4‐cyanopyridine (Entry 8, Table 2). Increasing the electron‐donating or ‐withdrawing strength of the substituent at C3 reduced the percentage yield of pyridination relative to 4‐CP.

**Table 2:** Scope of Cyanopyridine Derivatives.

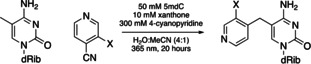					
Entry	X	Unreacted Nucleoside [%]^[b]^	Yield [%]^[b]^	Calculated Δ*G*_step2_^[c]^ [kcal mol^−1^]	Calculated Δ*G* ^≠^_step2_^[c,d]^ [kcal mol^−1^]
1	H	7	42	−1.1	14.1
2	Me	2	58	−4.8	11.5
3	F	60	24	5.1	14.0
4	Cl	70	20	3.4	–
5	Br	66	15	–	–
6	CN	82	4	10.4	21.7
7	OH	9	27	−0.6	36.0
8	NH_2_	95	0	−7.5	27.9

[a] All reactions were carried out using nucleoside (50 mM), 4‐cyanopyridine (300 mM) and xanthone (10 mM) in the solvent of MeCN and H_2_O (v/v 1/4, 0.5 mL total). The mixture was stirred under 365 nm UV light at room temperature for 20 hours under argon. [b] Percentage recovery of 5mdC and yields were calculated by integrating the UV absorbance of LCMS peaks relative to 2′‐deoxyinosine internal standard. [c] The calculated values were obtained at the B3LYP‐D3/6‐31G(d)//ωB97X‐D/6‐311++G(d,p) level of theory. [d] The kinetic barrier for the direct proton transfer from the methyl group of 5mdC to the pyridine: see section 4.3.2B. in Supporting Information for further details.

DFT calculations were carried out and showed that the variation in percentage yield between cyanopyridine substrates is mainly determined by the proton transfer step (Step 2, Figure 3) of the overall pyridination process (Section 5.4, Supporting Information). The experimentally measured yield of the reaction goes down as step 2 of the reaction becomes less thermodynamically favourable (Entries 1–6, Table 2), except in cases for which the free energy barrier for the reaction is relatively high (Entries 7–8, Table 2).

**Figure 4:**
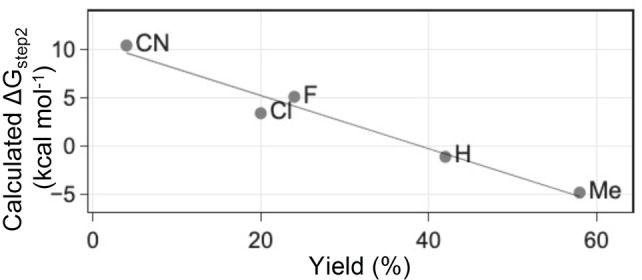
Calculated Δ*G*_step2_ vs. experimental yield plot for entry 1–4 and 6 in Table 2.

Groups that withdraw charge from the ring system make the ring more positive which in turn makes the acceptance of an extra proton by the pyridine substrate in step 2 thermodynamically less favourable. The correlation between the thermodynamic results (Δ*G*_step1_ and Δ*G*_step2_) and the electronic nature of the pyridine rings was explored through comparisons with experimental Hammett sigma constants (σ_x_).^[26]^ σ_x_ is a collective of the substituents’ ability to withdraw or donate electrons from the reaction site. Δ*G*_step2_ and σ_x_ are correlated by a positive linear trend (Figure S44C, Supporting Information). For cyanopyridines with electron withdrawing substituents (σ_x_>0.2), as Δ*G*_step2_ increases, we observed a decrease in percentage yield for the pyridination reaction (Figure 4).

Substrates with the ability to form hydrogen bonds are kinetically unfavourable in step 2. We performed conformational searching studies on cyanopyridine‐5mdC triplet dimers with Merck Molecular Force Field (MMFF) followed by DFT optimisations to transition states. Generation of 5mdC⋅ by direct deprotonation at the methyl C(sp^3^)−H bond is more kinetically favourable compared to deprotonation at the amine group due to the relatively high activation energy for intramolecular hydrogen‐atom transfer (Figure S40, Supporting Information). The Δ*G* ^≠^_step2_ for 3‐Me‐4‐CP (+11.5 kcal mol^−1^) is lower than Δ*G* ^≠^_step2_ for unsubstituted 4‐CP (+14.1 kcal mol^−1^) and may explain the increased percentage yield for this cyanopyridine (Entry 2, Table 2). The Δ*G* ^≠^_step2_ for 3‐hydroxy‐4‐cyanopyridine (3‐OH‐4‐CP) and 3‐amino‐4‐cyanopyridine (3‐NH_2_‐4‐CP) (+36.0 kcal mol^−1^ and 27.9 kcal mol^−1^ respectively) are both larger than Δ*G* ^≠^_step2_ for unsubstituted 4‐CP (+14.1 kcal mol^−1^) and may explain the decreased percentage yield for these electron‐donating substituents (Entries 7–8, Table 2).

**Figure 5:**
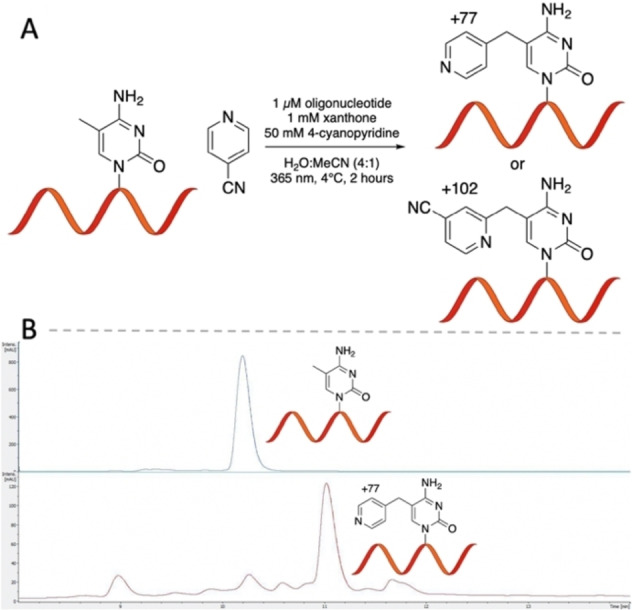
Pyridination of a 5mC‐containing DNA oligonucleotide. (A) Pyridination reaction Scheme with observed pyridination (+77) and Minisci radical addition (+102) reaction products. (B) Representative LCMS chromatogram of starting oligonucleotide (top) and pyridination reaction products (bottom).

We next sought to assess the pyridination reaction on DNA and RNA oligonucleotides. We began by establishing a pyridination protocol for the 12‐base oligonucleotide AGACCA5mCAACCA (5mC 12mer) (Figure 5A). Performing the reaction on single stranded oligonucleotides is compatible with both DNA and RNA sequencing library preparation and avoids unwanted charge transfer.^[27]^ The selectivity of pyridination is more challenging at the oligonucleotide level owing to the presence of many other potential C(sp^3^)−H targets. Furthermore, the steric and electronic factors of adjacent nucleobases place additional challenges on the reaction. The pyridination reaction conditions were modified to optimise conversion on oligonucleotides. Doubling the concentration of xanthone to 20 mM increased the yield of pyridination. Oligonucleotide reactions were performed at 4 °C. We found that carrying out the reaction at pH 7.1 lead to an efficient pyridination of 5mC with minimal off‐target reactivity (Figure 5B). 91 % conversion of the oligonucleotide occurred within 2 hours (Section 3.2.1, Supporting Information). Pyridination of 5mC accounted for 48 % of the observed products, another oligonucleotide reaction product was observed corresponding to an increase of *m*/*z* of +102 (Figure S26, Supporting Information). Based on previously reported radical additions to 4‐cyanopyridine, we hypothesise this is the Minisci radical addition product corresponding to addition of the 5mC‐methyl radical to the C2‐position of 4‐cyanopyridine.^[28]^ The Minisci product accounted for under 11 % of the observed rection product. When the pyridination reaction was performed on a 10mer oligonucleotide containing both 5mC and T (AGACCTAC5mCA), the overall yield of 5mC pyridination was over 5‐fold greater than that of T (46 % vs. 9 % respectively) (Section 3.2.2, Supporting Information). To verify the pyridination and Minisci substitution products occurred on both 5mC and T, the pyridination reaction was carried out on a 54mer oligonucleotide which was then subject to DNA degradase plus digestion and the resulting nucleoside mixture analysed by LCMS. Nucleoside pyridination product peaks 319.29 (5mdC+77), 320.31 (dT+77), 344.43 (5mdC+102), 345.30 (dT+102) confirm that these nucleosides were modified under the pyridination conditions (Figure S25, Supporting Information).

We next investigated the pyridination chemistry with an RNA 10mer oligonucleotide (agaccuac[m5c]a). RNA lacks the methyl group of the T nucleobase, which is replaced by U, this removes one of the selectivity constraints, however RNA is more chemically labile than DNA. The pyridination chemistry is specific for the methyl group of m5C in RNA and no side products at the other canonical bases were observed by LCMS analysis (Figure S29, Supporting Information). After 2 hours under the optimized pyridination reaction conditions, pyridination at m5C accounted for 18 % of the observed reaction products. The Minisci radical addition product accounted for 19 % of the observed reaction products (Section 3.2.3, Supporting Information).

A method to chemically enrich m5C in mRNA would be of value. Milder BS‐seq conditions are used to detect m5C on RNA, yet it is still destructive and the conditions do not result in complete conversion of C to U.^[29]^ RNA BS‐seq is therefore inadequate for the detection of m5C on low abundance RNA species or at m5C sites with a low methylation frequency. m5C RNA immunoprecipitation (m5C‐RIP) is the only other method that directly maps m5C.^[30]^ Immunoprecipitation methods such as m5C‐RIP can only tolerate low stringency washing conditions as RNA is non‐covalently bound. This increases the recovery of non‐specifically bound RNA and leads to a high level of noise in sequencing data.^[31]^ The major chemical modifications in mRNA are m5C (0.1–0.4 % m5C/C),[^29^, ^32^] N6‐methyladenosine (m6A) (0.1–0.4 % m6A/A),^[33]^ and pseudouridine (Ψ) (0.1–0.25 % Ψ/U).^[34]^ Pseudouridine contains no methyl group therefore it is expected to be unreactive to the pyridination chemistry. Pyridination of RNA nucleosides using HaloLig‐CP was specific for m5C (27 %) over m6A (0 %) (Figures S29–30, Supporting Information). Low levels of m6A demethylation (2 %) were observed, likely due to formation of N6‐hydroxymethyladenosine via an α‐amino radical intermediate.^[14]^ The demethylation of m6A to A would not affect the chemical enrichment of m5C. The high selectivity of the pyridination reaction towards m5C in RNA with HaloLig‐CP suggests it has a good potential to enrich m5C in the context of genomic mRNA.

**Figure 6:**
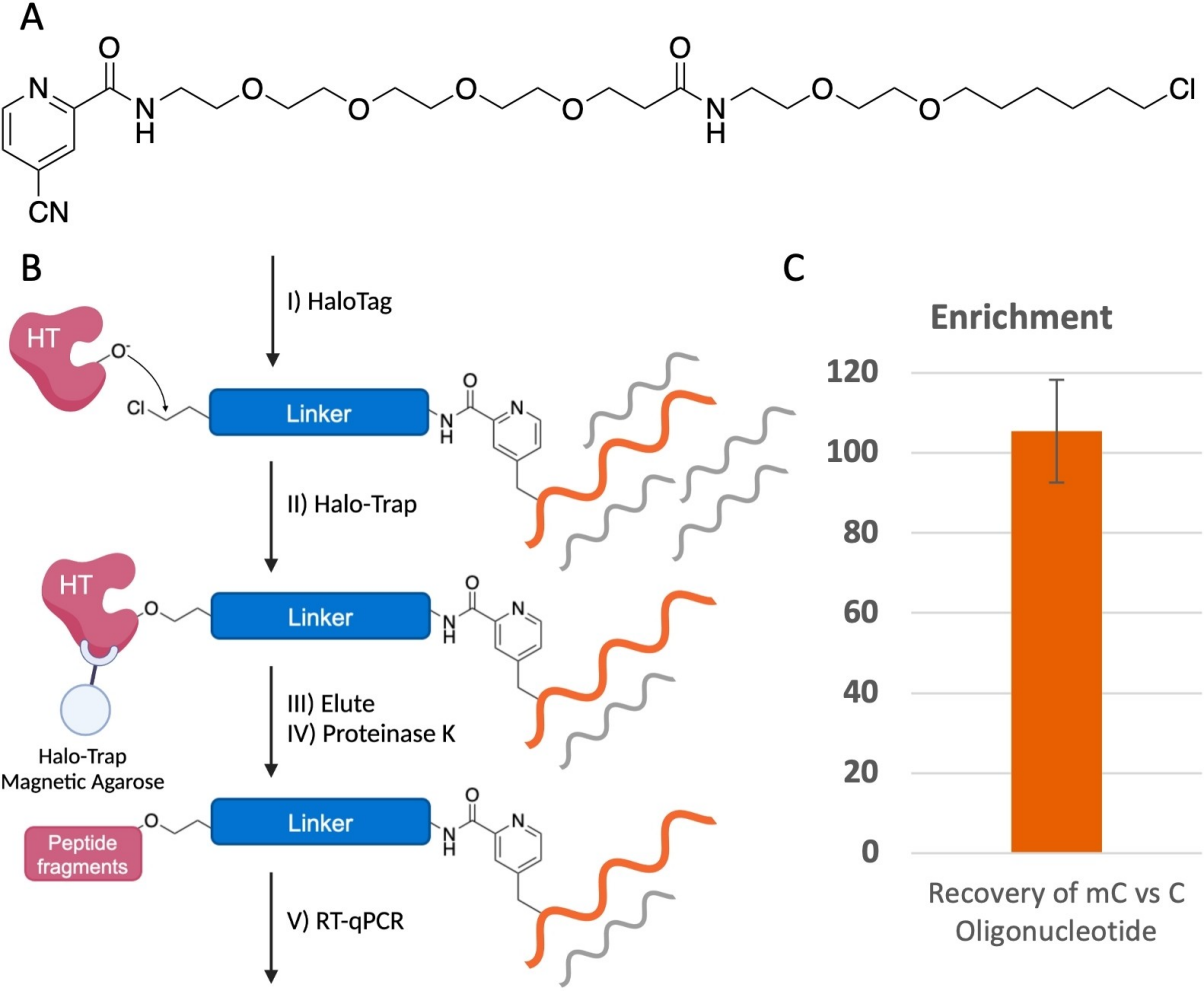
HaloTag‐based pull‐down strategy for the enrichment of m5C‐containing RNA oligonucleotides. (A) Structure of HaloTag‐reactive HaloLig‐CP. (B) Pull‐down procedure involving HaloTag conjugation following selective m5C chloroalkane functionalisation via pyridination. HaloTag‐bound RNA is then enriched using Halo‐Trap Magnetic Agarose. (C) Recovery of a 67nt m5C‐containing RNA oligonucleotide relative to a 66nt C‐containing RNA oligonucleotide.

The specific nature of the pyridination reaction to modify the methyl group of m5C on RNA as well as the broad substrate scope of cyanopyridine derivatives suggested the chemistry can be expanded to enable chemical enrichment of m5C in RNA. We designed a chloroalkane‐containing cyanopyridine (HaloLig‐CP) that can form a covalent bond to the HaloTag protein tag (Figure 6A). HaloTag rapidly forms a covalent adduct with chloroalkane substrates at low micromolar concentrations.^[35]^ Both C‐CN and Minisci substitution pathways result in the attachment of an enrichable handle at m5C. When the pyridination reaction with HaloLig‐CP was carried out on 5mC 12mer, 66 % of the oligonucleotide products were functionalised with HaloTag‐reactive chloroalkane (Figure S32, Supporting Information). 18 % of the reaction product corresponded to oxidation of 5mC to 5fC. This does not significantly hinder the application of the pyridination chemistry for enrichment, as it is still highly selective for m5C.

We confirmed that commercially available HaloTag‐GST fusion protein can conjugate to a chloroalkane‐functionalised oligonucleotide by protein LCMS (Figure S34, Supporting Information). Cyanopyridines with shorter chloroalkane chains were poor substrates for HaloTag (Figure S35, Supporting Information). The crystal structure of DhaA, the protein HaloTag is derived from, shows that the enzyme active site is located within a narrow binding pocket.^[36]^ Computational models suggest this site measures approximately 15 Å from the surface of the protein, which suggests chloroalkane ligands longer than four methylene groups would be sufficient to bind the protein.^[35]^ A possible reason for the poor reactivity is that the oligonucleotide adduct may interfere with HaloTag binding. The surface of the HaloTag protein is negatively charged therefore binding to an oligonucleotide substrate could be significantly influenced by electrostatic interactions.^[37]^

Affinity‐based enrichment procedures for HaloTag provide a means to enrich m5C‐containing oligonucleotides. We began the enrichment procedure by subjecting a mixture of a 67nt m5C‐containing RNA oligonucleotide (RNA‐M) and a 66nt C‐containing RNA oligonucleotide (RNA‐C) with a 10‐fold excess of heparin to the pyridination reaction. The chemical treatment of RNA during RNA BS‐seq causes extensive RNA degradation and reduces the recovery of complementary DNA (cDNA) following reverse transcription.[^4^, ^38^, ^39^, ^40^, ^41^] Quantitative reverse transcription polymerase chain reaction (qRT‐PCR) of RNA‐M and RNA‐C immediately following pyridination consistently recovered greater than 60 % of oligonucleotide. Loss of oligonucleotide could be a result of decrease in reverse transcription efficiency as well as oligonucleotide degradation. The chloroalkane‐functionalised RNA was then conjugated to HaloTag‐GST and enriched using Halo‐Trap Magnetic Agarose (Figure 6A & Section 4.4, Supporting Information). A 105‐fold enrichment of RNA‐M over RNA‐C was achieved, as determined by qRT‐PCR (Figure 6B). This compares favourably with previously reported RNA m5C enrichment methods such as m5C‐RIP, that showed 20‐fold enrichment for m5C.^[42]^ The specificity of the pyridination reaction for the methyl group of m5C in RNA and the ability to enrich an m5C‐containing RNA oligonucleotide suggests a future potential for this methodology to improve the detection and study of m5C in a biological context.

## Conclusion

In summary, we have shown that the C(sp^3^)−H bond of the methylcytosine methyl group can be selectively modified by triplet photosensitised catalysis. The reaction shows promise for the selective manipulation of m5C in RNA, as demonstrated by the chemical enrichment of m5C‐containing RNA oligonucleotides. Chemical‐based enrichment of m5C would be of particular value for the detection of m5C in low abundance mRNA species where RNA BS‐seq is too destructive and insensitive.^[6]^

## Conflict of interest

S.B. is an advisor and shareholder of Biomodal Ltd. (formerly called Cambridge Epigenetix Ltd.), Inflex Ltd. and Elyx Ltd. A patent application has been filed based on some of the work described in this manuscript.

1

## Supporting information

As a service to our authors and readers, this journal provides supporting information supplied by the authors. Such materials are peer reviewed and may be re‐organized for online delivery, but are not copy‐edited or typeset. Technical support issues arising from supporting information (other than missing files) should be addressed to the authors.

Supporting Information

## Data Availability

The data that support the findings of this study are available in the supplementary material of this article.
